# The Influence of Dengue Virus Serotype-2 Infection on *Aedes aegypti* (Diptera: Culicidae) Motivation and Avidity to Blood Feed

**DOI:** 10.1371/journal.pone.0065252

**Published:** 2013-06-03

**Authors:** Rafael Maciel-de-Freitas, Gabriel Sylvestre, Mariana Gandini, Jacob C. Koella

**Affiliations:** 1 Laboratório de Transmissores de Hematozoários, Instituto Oswaldo Cruz, Fiocruz, Brazil; 2 Laboratório de Imunologia Viral, Instituto Oswaldo Cruz, Fiocruz, Brazil; 3 Institut de Biologie, Université de Neuchâtel, Switzerland; Institut Pasteur, France

## Abstract

**Background:**

Dengue virus (DENV) is transmitted by *Aedes aegypti*, a species that lives in close association with human dwellings. The behavior of DENV-infected mosquitoes needs further investigation, especially regarding the potential influence of DENV on mosquito biting motivation and avidity.

**Methodology/Principal findings:**

We orally challenged 4–5 day-old *Ae. aegypti* females with a low passage DENV serotype -2 (DENV-2) to test whether the virus influences motivation to feed (the likelihood that a mosquito obtains a blood-meal and the size of its blood meal) and avidity (the likelihood to re-feed after an interrupted first blood-meal). To assay motivation, we offered mosquitoes an anesthetized mouse for 2, 3, 4 or 5 minutes 7 or 14 days after the initial blood meals and measured the time they started feeding. 60.5% of the unexposed mosquitoes fed on the mouse, but only 40.5% of the positive ones did. Exposed but negative mosquitoes behaved similarly to unexposed ones (55.0% feeding). Thus DENV-2 infection decreased the mosquitoes’ motivation to feed. To assay avidity, we offered the same mosquitoes a mouse two hours after the first round of feeding, and we measured the time at which they started probing. The exposed (positive or negative) mosquitoes were more likely to re-feed than the unexposed ones and, in particular, the size of the previous blood-meal that kept mosquitoes from re-feeding was larger in the exposed than in the unexposed mosquitoes. Thus, DENV-2 infection increased mosquito avidity.

**Conclusions/Significance:**

DENV-2 significantly decreased the mosquitoes’ motivation to feed, but increased their avidity (even after taking account the amount of blood previously imbibed). As these are important components of transmission, we expect that the changes of the blood-feeding behaviour impact the vectorial capacity *Ae. aegypti* for dengue.

## Introduction

Dengue fever is a mosquito-borne disease caused by infection with one of the four serotypes of dengue viruses (DENV) in the family *Flaviviridae*. Dengue occurs mainly in tropical countries, where around 2.5 billion people live at risk of infection and 50–100 million new cases occur annually [1]. In the Americas, dengue transmission occurs in almost all metropolitan areas where its primary vector, the mosquito *Aedes aegypti*, is present. These mosquitoes are commonly found in high numbers in urban areas, and females often rest inside homes and lay eggs in artificial containers near human dwellings [2]–[4].

Dengue transmission and epidemiology are influenced by the parameters underlying the mosquito’s vectorial capacity, such as its population density, its probability of daily survival, its biting behavior and the parasite’s extrinsic incubation period [5]. Thus, to understand the epidemiology of dengue, we must understand how the mosquito-dengue interaction influences these epidemiologically relevant traits. For instance, dengue infection reduces *Ae. aegypti* female survival and fecundity from the third clutch of eggs [6]. Studies on the biting behavior of dengue-infected mosquitoes are scarce, and these give conflicting results. Using mosquitoes from long-established laboratory colonies, Putnam and Scott [7] found no evidence that dengue virus serotype 2 (DENV-2) influences the mosquito’s feeding behavior (i.e. biting rate). On the other hand, Platt et al. [8] observed that the time required for blood feeding and the time spent probing are longer in dengue-infected mosquitoes than in uninfected individuals.

We were interested in the influence of dengue-infection on two aspects of the mosquito’s biting behavior. First, to confirm and expand on Platt et al.’s [8] results, we considered what we call the *motivation* of the mosquito to feed: the likelihood that they feed (within a limited period of time) and the time required to start feeding. Second, we measured the *avidity* of the mosquito: the probability that a partially fed mosquito feeds a second time to complete its blood-meal. We further considered whether the effect of dengue-infection on these two aspects of blood-feeding behavior vary with the mosquito’s age.

## Methods

### Mosquitoes

We used the Paea strain of *Ae. aegypti*, a laboratory colony initiated with mosquitoes collected in French Polynesia in 1994 and maintained in the laboratory for about 400–450 generations. This strain is highly susceptible to oral dengue infections and is routinely used as a control in vector competence experiments under laboratory controlled conditions [9]. Preliminary results showed 100% infection of Paea strain 14 days post-infection (dpi) using the DENV-2 strain 16681 (GS, unpublished data).

Larvae were reared on yeast extract and raised in plastic basins at 25±3°C. Adults were maintained at 27±2°C, 75±5% relative humidity and 12–12 h light-dark photoperiod in cages of 45 cm^3^. They were fed *ad libitum* with cotton soaked with 10% sucrose.

### Virus

Dengue virus type 2 strain 16681 [10] with 6 passages on C6/36 cells was used for virus stock preparation as described elsewhere [11]. Briefly, *Ae. albopictus* cell clone C6/36 (CRL-1660, ATCC) was maintained at 28°C in Dubelcco’s modified Eagle Medium (Gibco, Life Technologies) with sodium bicarbonate and supplemented with 5% fetal bovine serum (FBS), 1% penicillin-streptomycin-glutamine (Gibco), 0,5% non-essential amino acids (Gibco) and 10% tryptose phosphate (Sigma). C6/36 cell monolayers were infected with DENV-2 and cell culture supernatants were harvested 8 days later. A purified DENV-2 stock was obtained by ultracentrifugation at 100,000 g for 1 h and set to a final volume 20 times smaller than initial [12]. Titration was performed in C6/36 cells using a standard TCID_50_ (50% tissue infective dose) assay as described elsewhere [13]. Uninfected flasks were maintained, purified and used as negative control (MOCK).

### Oral Infection of Mosquitoes with DENV-2

Four or five days after emergence, 60 females per cage were put into small (12 cm height, 15 cm diameter) cylindrical plastic cages, with no access to sugar. About 36 h later, they were offered a DENV-2 infectious blood meal. One ml of supernatant of infected cell culture was added to 2 ml of washed rabbit erythrocytes to prepare the infectious blood-meal, which was heated to 37°C and provided to the mosquitoes in an artificial membrane feeding apparatus [14]. Mosquitoes were allowed to feed for 25 min on infectious blood that contained a viral titre of 2×10^8^ TCID_50_. The same procedure and apparatus were used to feed control mosquitoes, except that these received a non-infectious blood meal, with 1 ml of culture medium replacing the viral supernatant. We conducted four infection assays.

### Experimental Design

The females that were fully engorged were isolated in labelled cylindrical plastic tubes (6.5 cm height, 2.5 cm diameter) containing moistened cotton overlaid with filter paper as substrate for oviposition on the bottom and closed on the top with mosquito netting. Half of the mosquitoes were assayed for their blood-feeding behaviour 7 days after their infective blood-meal, the other half 14 days after their blood-meal.

### Feeding Behaviour

We assayed two aspects of blood-feeding behaviour. (i). *Motivation.* Our two measures of a mosquito’s motivation to blood-feed were the likelihood that it attempted to feed within a given amount of time, and the time it started feeding. On each day of the assay (7 and 14 days after their infective blood-meal), mosquitoes were given access to an anesthetized mouse for 2, 3, 4 or 5 minutes, and we recorded the time each mosquito started ingesting blood. (ii) *Avidity*. Our measure of avidity was the likelihood that a mosquito attempted to re-feed, as a function of the size of its earlier blood-meal. Around two hours after the first blood meal, the mosquitoes were offered the same anesthetized mouse as in the first round of feeding, and we assayed whether the mosquitoes started probing (i.e., inserted the proboscis into the mouse) within five minutes. We removed the mosquitoes when they started probing, so that they were not able to top up their blood-meal. Note that varying the amount of time available to blood-feed during the first round of blood-feeding generated the variability of blood-meal size required to measure avidity (see Results).

Immediately after assessing female avidity, i.e., after the second round of blood-feeding, mosquitoes were killed by freezing.

Both assays of feeding behaviour were done without knowledge of the infection status or age of the mosquitoes.

### Blood Meal Size

We estimated the amount of blood ingested by the mosquitoes with a method developed by Briegel et al. [15]. Briefly, the abdomens of individual mosquitoes were ground in 1 ml of Drabkin`s solution, and the amount of haemoglobin in the solution was estimated by the optical density measured in a spectrophotometer at 540 nm.

### Immunofluorescence Assays (IFA)

DENV detection was performed by indirect fluorescent antibody test (IFAT) using serotype-specific monoclonal antibodies [16].

### Statistical Analyses

For analysis, mosquitoes were separated into three groups: unexposed controls, which had not fed on DENV-2 infectious blood; positive mosquitoes, which had been blood-fed with a DENV-2 infective blood-meal and in which IFA detected DENV particles; and exposed but negative mosquitoes, which had been fed with infective blood but were negative in IFA assays.

*Motivation*. We analyzed the likelihood of a mosquito biting in the first feeding with a logistic analysis that included age at assay (categorical variable: 7 or 14 dpi), infection status (categorical variable: control, infected and exposed but negative), their interaction and, as a confounder, the time available for feeding (ordinal variable), The time required to start feeding was analyzed with a survival analysis containing the same variables apart from the time available for feeding (as this is accounted for with the censor of the survival analysis). We present the parametric analysis with the distribution that provided the best fit; other distributions and a non-parametric analysis (proportional hazards) gave similar results.

We analyzed bloodmeal size with an anova of optical density (representing the amount of blood that was ingested in the first feeding) including, age at assay and infection status.

*Avidity*. To address the factors influencing mosquito avidity during a second feeding, we first confirmed that the time available for blood-feeding during the first round of feeding did indeed influence the blood-meal size. We analyzed avidity with a logistic analysis of the likelihood that mosquitoes attempted to re-feed as a function of age at assay, infection status, the optical density and the interactions with infection status. As for our measure of avidity we were mainly interested in the effect of infection in the relationship between blood-meal size and the likelihood of re-feeding, we only analyzed mosquitoes that had attempted to feed during the first round.

In all analyses, we had first included wing length but then excluded it from the presented results as it had very little impact on our measures of blood-feeding behavior. All analyses were done with JMP 10.0.

### Ethics Statement

Mosquito blood-feeding on anesthetized mice was authorized by Fiocruz Ethical Committee (CEUA 007/009).

## Results

We assayed the blood-feeding behavior of 119 mosquitoes 7 days after infection and 81 mosquitoes 14 dpi. On 7 dpi, 40 females were not exposed to DENV-2, 48 were positive and 31 were exposed but were negative in further IFI assays. On 14 dpi, 36 mosquitoes were not exposed, 36 were positive and 9 were exposed but negative.

**Figure 1:**
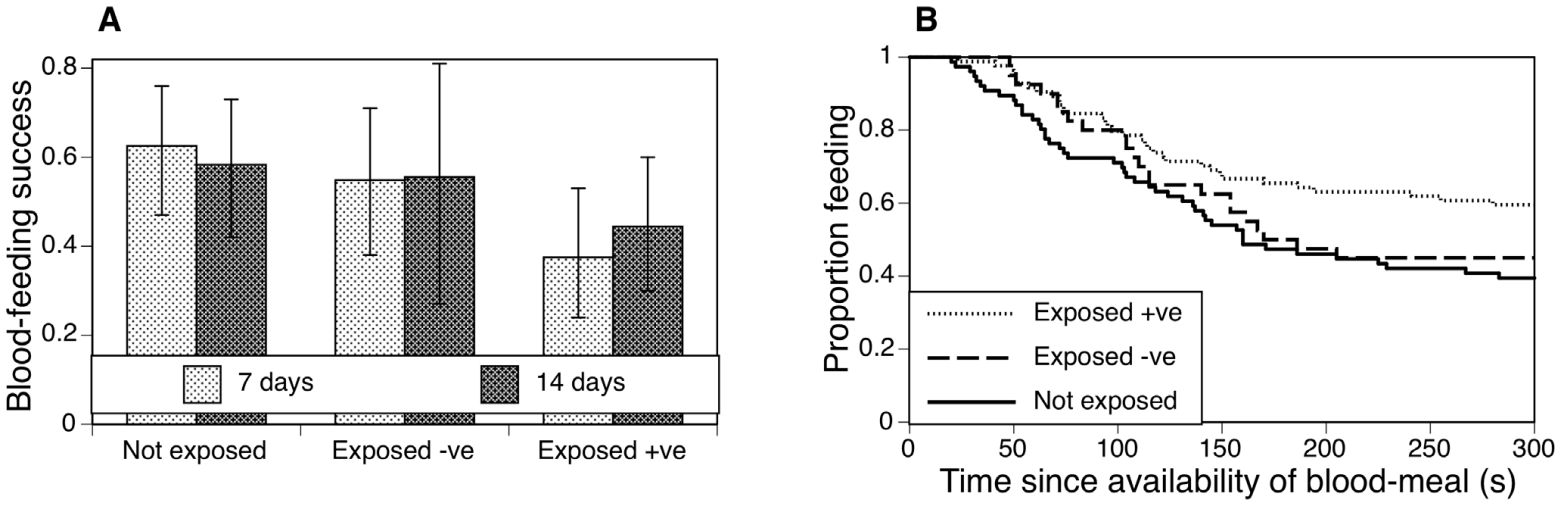
Effect of infection status on motivation of mosquitoes to blood-feed. A. Proportion of mosquitoes that obtained blood within a fixed amount of time, as a function of age at assay and infection status. B. Time required for mosquitoes to start feeding as a function of infection status (The mosquitoes of the two ages at exposure were pooled to simplify the graph).

*Motivation to feed (first feed):* 102 (51%) mosquitoes blood fed during the first round of feeding. The likelihood that mosquitoes attempted to feed within the time available to them was highest for the unexposed mosquitoes (46 out of 76) and lowest for the positive ones (34 out of 84); the exposed but negative mosquitoes (22 out of 40) behaved similarly to the unexposed ones. The effect of infection appeared to be greater in the young than in the old mosquitoes (Fig. 1A), but this interaction was far from statistically significant (Table 1). The time available for feeding had little impact (Table 1). A similar pattern held for the time to start feeding (Table 1): uninfected mosquitoes started feeding more rapidly than either exposed but negative or positive ones (Fig. 1B), and age had little influence.

**Table 1:** Statistical analyses of the mosquitoes’ motivation to blood-feed.

		Feeding success		Time to start feeding	
Source	df	X^2^	*P-value*	X^2^	*P-value*
Time available	3	0.24	0.971	−	–
Age	1	0.02	0.894	0.06	0.813
Infection status	2	6.16	0.045	7.71	0.021
Age×infection	2	0.48	0.787	2.07	0.355

Feeding success (whether a mosquito obtained blood during the first round of feeding) was analysed with a logistic analysis; time to start feeding was analysed with a survival analysis (Frechet distribution).

Among the mosquitoes that attempted to feed, the blood-meal size increased with the amount of time available for feeding (F_3,92_ = 6.5, p<0.001) from an optical density of 0.43 (±0.053 s.e.) after 2 minutes to an OD of 0.75 (±0.061 s.e.) after 5 minutes, confirming that our experimental design constrained blood-meal size. Blood-meal size was greatest in positive mosquitoes, intermediate in unexposed but negative ones and smallest in unexposed ones, but this effect was not statistically significant (F_2,92_ = 2.3, p = 0.109). Blood-meal size decreased with age (F_1,92_ = 17.2, p<0.001), and the effect of age was influenced by infection status (interaction age×infection: F_2,92_ = 5.5, p = 0.005).

**Figure 2:**
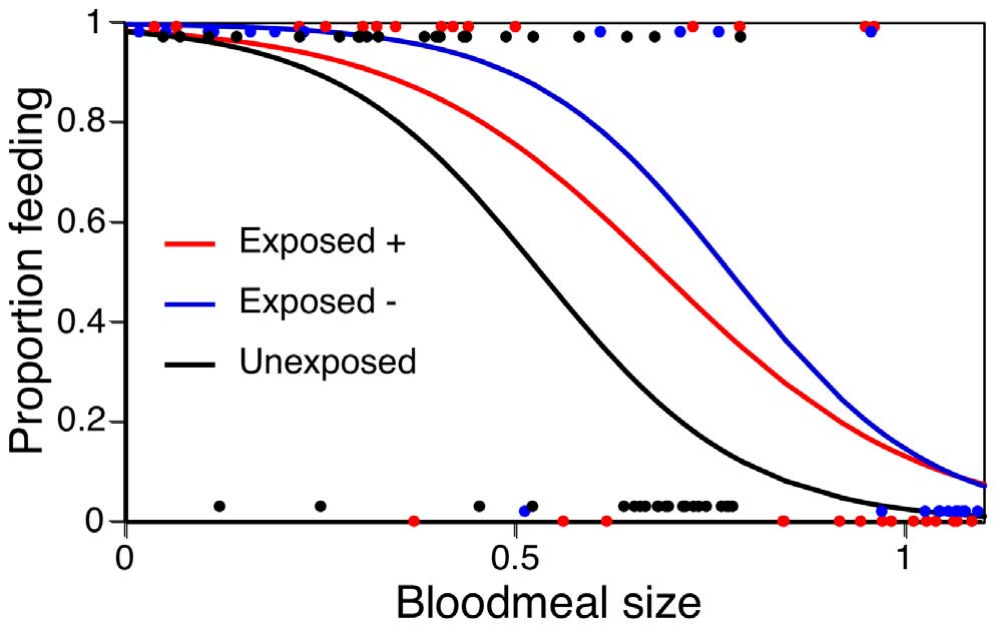
Effect of infection status on avidity of mosquitoes. Proportion of mosquitoes that attempted to re-feed two hours after their first feeding attempt, as a function of their infection status and amount of blood they had imbibed earlier (measured as optical density). Dots represent individual mosquitoes and are set to 1 if they attempted to feed within five minutes and to 0 if they did not. Lines show the results of logistic regressions through the dots.

*Avidity (second feed):* Mosquitoes that had not fed during the first round of feeding opportunity were considerably more likely to feed during the second round (85% vs 51%; χ^2^_1_ = 27.0, p<0.001). For the following results, we used only the mosquitoes that had fed during their first feeding round. Older mosquitoes were more likely to re-feed (74% vs 63%), the effect of age was modified by infection status, and as expected, the more blood mosquitoes had imbibed during their first feed, the less likely they were to bite during a second round of feeding (Table 2). Most relevant for this paper is that infection modified avidity (Table 2). Although the main effect of infection was not significant, its interaction with blood meal size was: the blood meal size that kept 50% of the mosquitoes from attempting to re-feed was greater for positive (optical density: 0.68) and for exposed but uninfected mosquitoes (0.77) than for control mosquitoes (0.53) (Fig. 2).

**Table 2:** Statistical analyses of the mosquitoes’ avidity.

Source	df	Χ^2^	*P-value*
Age	1	11.9	<0.001
Blood-meal size (OD)	1	67.5	<0.001
Infection status	2	2.7	0.264
Age×infection	2	14.0	<0.001
Blood-meal×infection	2	9.8	0.008

Logistic analysis of the amount of blood previously imbibed (measured as optical density, OD), the mosquito’s age at the assay and its infection status on the mosquito’s likelihood to bite during a second round of blood feeding.

## Discussion

Dengue infection affected the biting behaviour of the mosquito vector, *Ae. Aegypti*, reducing motivation to feed, but increasing the desire to re-feed (at a given amount of blood previously imbibed). We had expected that dengue would change behaviour in a way that could be interpreted as manipulation to increase transmission, similar to other parasite-vector interactions [17], most notably in a series of papers on malaria and mosquitoes [18]–[24]. The patterns we observed, however, only partially support the idea. On the one hand, the probability to feed to top up a previous bloodmeal (the avidity) was indeed increased by dengue virus infection, which is expected to increase transmission, in particular if the subsequent bites are on different hosts [25], [26]. On the other hand, infection decreased the likelihood that the mosquito attempted to feed within a restricted time (its motivation) during the first round of feeding and increased the time required to start feeding.

Like other arboviruses, dengue virus can invade the mosquito’s brain, which may modify its physiology and metabolism and thus change life-history traits directly involved in vectorial capacity [8], [27]. For instance, two studies considered the biting rate of dengue-infected *Ae. aegypti*, but gave conflicting results. Using mosquitoes from long-established laboratory colonies, Putnam and Scott [7] found no evidence that DENV-2 influences the mosquito’s feeding behavior (i.e. biting rate). IN contrast Platt et al. [8] observed that the time required for blood feeding and the time spent during probing was longer in dengue-infected mosquitoes than in uninfected individuals. Other life-history traits are also changed by dengue virus; *Ae. aegypti* females infected with DENV-2, for example, increase their locomotor activity by up to about 50% [28],possibly enhancing their chance of finding a suitable host and thus increasing their biting rate. However, all three of these studies [7], [8], [28] infected mosquitoes by intrathoracic inoculation, which is an invasive and unnatural mode of transmission that has important consequences on the dynamics of virus-vector interactions [29].

After simplifying a previously developed theoretical model, Luz and co-workers examined the potential impact of increases in biting activity on dengue transmission [27]. When no difference was assumed between the biting rates of infected and uninfected *Ae. aegypti* mosquitoes, the model predicted annual dengue epidemics of a relatively constant size. When assuming that the biting rate of infected *Ae. aegypti* was increased, the number of infections was increased as well. For example, when assuming a 50% increase in the biting rate of dengue-infected mosquitoes, the percent increases in the number of primary and secondary dengue infections were 3.8% and 6.5%, respectively [27].

Mosquito age had little influence on the motivation of the mosquitoes’ blood-feeding behaviour, corroborating other reports on the impact of aging on mosquito biting behaviour [30]. When comparing the feeding behaviour of dengue infected and uninfected individuals, Platt et al. [8] observed mosquito aging did not statistically affect seven parameters related to mosquito feeding behaviour, including the total feeding time and the mean probing time. In addition, while in another study the length of time to first bite, the number of bites and blood meal acquisition were all strongly influenced by an interaction between the mosquitoes’ age and *Wolbachia* infection, there was no effect of age in uninfected mosquitoes [31]. A complementary report showed that aging did not influence the time required to insert insect`s mouthparts into a host, start probing, blood meal acquisition or the number of bites [32]. On the other hand, our study suggests that age does influence other aspects of the mosquito’s biting behaviour, in particular the likelihood that mosquitoes re-feed once they have imbibed less than a full blood-meal.

Our study suffers from possible caveats, for we used a nearly two-decade-old *Ae. aegypti* laboratory strain and non-human blood to infect mosquitoes. We used the Paea strain to maximize the success of infection, for this strain presents high susceptibility to DENV [9]. Furthermore, due to ethical and logistical concerns we used blood meals composed of cultured virus mixed with animal blood presented to mosquitoes across a skin-simulating membrane. A more natural but ethically controversial approach would be to infect mosquitoes directly on the arms of dengue patients with viraemia [33].

It should be noted that our measures of motivation and avidity may be correlated: the mosquitoes that previously imbibed more blood are more motivated than those that took up less blood. However, we believe that this possible correlation has no influence on our results or interpretation. First, we constrained the size of the blood-meal by giving the mosquitoes variable amounts of time to feed during the first round. Second, mosquitoes that had not fed during the first round of feeding opportunity were more likely to feed during the second round, suggesting that it was not a lack of motivation that had constrained feeding. Third, the important result about avidity is that infection changed the relationship between blood-meal size and the likelihood of re-feeding; this interaction is not a simple consequence of differences of motivation.

Overall, our study suggests that DENV-2 significantly decreases the mosquitoes’ motivation to feed, but increases their avidity. As these behaviours are important components of transmission, we expect that the changes of the blood-feeding behaviour impact the vectorial capacity *Ae. aegypti* for dengue. However, the two components change vectorial capacity in opposite ways; whether transmission is increased overall depends on details of the feeding ecology, e.g. how frequently mosquitoes feed twice. Furthermore, in contrast to other parasite-vector associations, it is unlikely that the changes are a result of evolutionary pressures to manipulate the mosquito.
